# Combination of Photon and Carbon Ion Irradiation with Targeted Therapy Substances Temsirolimus and Gemcitabine in Hepatocellular Carcinoma Cell Lines

**DOI:** 10.3389/fonc.2017.00035

**Published:** 2017-03-13

**Authors:** Sarah Dehne, Clarissa Fritz, Stefan Rieken, Daniela Baris, Stephan Brons, Thomas Haberer, Jürgen Debus, Klaus-Josef Weber, Thomas E. Schmid, Stephanie E. Combs, Daniel Habermehl

**Affiliations:** ^1^Department of Radiation Oncology, University Hospital of Heidelberg, Heidelberg, Germany; ^2^Heidelberg Ion Beam Therapy Center (HIT), Heidelberg, Germany; ^3^Department of Radiation Oncology, Klinikum rechts der Isar, Technische Universität München, Munich, Germany

**Keywords:** carbon ion irradiation, hepatocellular carcinoma, Temsirolimus, Gemcitabine, combined modality treatment

## Abstract

**Background:**

This work investigates on putative cytotoxic effects in four different hepatocellular carcinoma (HCC) cell lines after irradiation with photons or carbon ions in combination with new targeted molecular therapy using either Temsirolimus (TEM) or Gemcitabine (GEM).

**Methods and materials:**

The HCC cell lines HepG2, Hep3B, HuH7, and PLC were cultured and irradiated with photons or carbon ions at the Heidelberg Ion Beam Therapy Center using the raster-scanning method. For combination experiments, cell lines were first treated with Temsirolimus or GEM before irradiation. Cytotoxicity was measured by a clonogenic survival assay. The evaluation of the experiments and the obtained survival curves were based on the concept of additivity defined by Steel and Peckham.

**Results:**

The results for the combination of carbon ions and both tested systemic substances TEM and GEM showed independent toxicities in all four cell lines. Supra-additive effects were observed in PLC cells for photon irradiation combined either with TEM or GEM and in HuH7 cells for the combination of photons with TEM.

**Conclusion:**

Addition of targeted therapy substances Temsirolimus and GEM to photon irradiation showed additive cytotoxicity in HCC cell lines, whereas independent toxicities where reached by the combination of carbon ions to these substances. It can be assumed that combining 12C with systemic substances only has independent effects because heavy ions cause direct damage because of their high-LET character resulting in complex and clustered double-strand breaks. Nonetheless, further investigations are warranted in order to determine whether addition of systemic therapy allows a reduction of radiation doses in combination therapy. This could possibly lead to better responses and tolerances in patients with HCC.

## Introduction

Hepatocellular carcinoma (HCC) is the fifth most common cancer worldwide and makes up the second common cause of cancer-related death in males. In women, it is the seventh most common cancer worldwide and the sixth leading cause of death. Because of an increasing incidence of HCC and due to a rising incidence of hepatitis C and liver cirrhosis (1), it has gained clinical interest, especially in areas like Europe and North America, which have had low rates of the disease yet. HCC is difficult to treat, because at initial presentation the disease is multifocal or locally advanced. The challenge of every applied treatment is to preserve sufficient rest liver function.

At this point, there are several therapy modalities, mainly surgical excision or liver transplantation, which provide the most mature outcome data (2, 3). However, there is a limit to a surgical therapy for HCC, because patients often present with a poor liver function because of an underlying cirrhosis, macro-vascular tumor invasion or advanced stage of the disease. Furthermore, interventional treatments, e.g., transarterial chemo-embolization, radio-frequency ablation, and radiotherapy (RT) are available, but data for these modalities are still sparse and to date no randomized trials are available.

In the past, RT only played a minor role in the treatment of liver malignancies, because of the livers low tolerance to radiation and the challenge to deliver high-dose irradiation to the target while sparing the uninvolved tissue (4). Due to several technological advancements such as highly conformal RT and particle-beam therapy (PBT), there is a more precise irradiation application to the tumor and a better surveillance of the beam while treatment (5, 6). In 1960s, irradiation with heavy ions has been launched in clinical practice and is now established for several cancer modalities. There is a clear physical advantage of particle beams—compared to photon irradiation—that consists in a deeper dose gradient due to an inverted dose profile provided by a spread-out Bragg peak. Carbon ion beams show a higher relative biological effectiveness (RBE), which therefore can overcome relative radio-resistance induced by hypoxia through induction of clustered DNA double-strand breaks (DSBs). This bears the hope of an improvement in treating HCC and other tumor entities (7).

The application of systemic chemotherapy to HCC has had limited impact on treatment of HCC. Only the multi-kinase inhibitor Sorafenib has been approved as an agent for standard therapy of advanced HCC (8). Nevertheless, newer targeted biological therapies like Temsirolimus (TEM) interfering with crucial molecular pathways in hepatocarcinogenesis are investigated and need to be evaluated in the future (9). Another systemic drug is Gemcitabine (GEM) showing promising results in different trials with hepatobiliary tumor entities but needs to be tested explicitly for HCC (10–12). This work is concerned with the evaluation of two systemic drugs, TEM, and GEM in four different HCC cell lines, especially in combination with photon and carbon ion (^12^C) irradiation.

## Materials and Methods

### Cell Culture

Four human HCC cell lines, Hep3B, HepG2, PLC, and HuH7 were used. Hep3B, HepG2, and PLC had been obtained from the American Type Culture Collection (ATCC, Manassas, VA, USA), whereas HUH7 had been obtained from the Japanese Collection of Research Bioresources Cell Bank (JCRB Cell Bank, Japan). HuH7, Hep3B, and PLC were grown in Dulbecco’s Modified Eagle Medium (Biochrom, Berlin, Germany), whereas HepG2 was maintained in RPMI 1640 medium (Biochrom, Berlin, Germany). Both media were supplemented with 10% heat-inactivated fetal bovine serum (Biochrom, Berlin, Germany) and 1% penicillin-streptomycin (Invitrogen, Darmstadt, Germany). The cells were stored lying flat in 175 cm^2^ tissue plastic flasks (Falcon, Becton-Dickinson Labware Europe, Le Pont de Claix, France) in an incubator at 37°C in humidified air with 5% CO_2_ and passaged weekly.

### Clonogenic Assay

The technique applied was the clonogenic assay, which allows the biological efficiency to be determined by measuring clonogenic cell death. In order to generate reliable results, every experiment was performed in triplets three times at independent days. At first, a defined and increasing amount of cells, adjusted to the increasing doses of irradiation and/or concentration of the drug under investigation, were seeded into 25 cm^2^ flasks (Falcon, Becton-Dickinson Labware Europe, Le Pont de Claix, France), filled with media and incubated for 24 h. After this 24-h incubation, the treatment could be performed and afterward the flasks were left for several days in the incubator. The number of days for incubation was nine. Finally, the flasks could be inspected under the microscope for surviving colonies, which are defined as cell accumulations containing at least 50 cells per colony. Colony counting was performed under the microscope with a threshold of minimum 50 cells per colony. From the determined surviving fractions, the plating efficiency (PE) and clonogenic survival were calculated. These results were used to generate survival curves, to define α- and β-parameters and to calculate RBE values. SigmaPlot (Systat Software GmbH, Erkrath, Germany) non-linear least-squares regression option was used to fit the linear-quadratic expression [−ln(*S*) = α*D + β*D2] to the resulting averaged survival fractions after normalizing plating efficiencies to the untreated samples (*S* is the number of surviving cell following a dose of *D*, and α and β are the respective sensitivity coefficients).

In order to assess the results in the combination experiments, four terms were adopted from the criteria of additivity published by Steel and Peckham (13). These terms are independent toxicity, additivity, supra-additivity, and sub-additivity. The term independent toxicity (additivity) is described as the sum of the single effects of each agent used in a combination experiment. The expected effect of combination of two agents can be presented in an isobologram. Do the results of a combination experiment exceed the expected sum effects of two single effects, what implies potentiating and/or radiosensitizing, the term supra-additivity (or synergism) is used. Sub-additivity due to inhibition or antagonisms describes a response, which is below the expected response of two single agents in combination.

### Irradiation

An X-ray irradiator (XRAD 320 Precision X-ray Inc., North Bradford, CT, USA) was used with 1.5 mm Al, 0.25 mm Cu, and 0.75mm Sn filtration. Irradiation took place with dose rates of 1.2 Gy/min with a voltage of 320 kV and a current of 20 mA. Irradiation of the cell monolayer was performed with doses of 2, 4, 6, and 8 Gy at room temperature.

Irradiation with ^12^C was conducted at the Heidelberg Ion-Beam Therapy center (HIT) using the raster-scanning method (14). To expose the cell monolayer to the middle of the extended Bragg peak, the ground of the flasks was arranged along a construction with a 3 cm acrylic shield and irradiated with a horizontal beamline using the raster-scanning technique. Single doses of 0.125, 0.5, 1, 2, and 3 Gy were delivered at the experiments with an averaged dose rate of 0.5 Gy per minute. The spread out of the Bragg peak was assured by positioning of a plexi glass device (30-mm thickness) in front of the cell culture flasks. LET of the C12 RT was between 122.36 and 136.92 MeV/u and the applied beam width measured 7.1–7.8 mm.

### Reagents

Gemcitabine (Gemzar^®^, dFdGem) was used in concentrations of 10, 30, 40, and 50 nM. For combination with RT (photon and ^12^C) concentrations of 10 and 30 nM were applied. Twenty-four hours after seeding the cells, the media was replaced with fresh media containing the reagents at the appropriate concentration and the cells were subsequently irradiated after 4-h incubation time. Temsirolimus (Torisel^®^, CCI-779) experiments were conducted on PLC and HuH7 cells with concentrations of 0.01, 0.1, 1, and 2 nM, while combination therapy with irradiation took place with a concentration of 0.1 nM. Experiments with Hep3B and HepG2 were conducted with 500, 750, 1,000, 1,500, and 2,500 nM. The subsequent combination therapy was performed with 750 nM of TEM.

Sensitivity to the anti-tumorigenic effect of both drugs was expressed as an IC_50_ value which represents the drug concentration causing 50% inhibition of the clonogenic survival. The half maximal inhibitory concentration of both substances was calculated for all cell lines.

### Statistical Analysis

Data were analyzed using SigmaPlot (Systat Software GmbH, Erkrath, Germany). Data are presented as the mean ± SE. Differences with a *p* < 0.05 were considered statistically significant. Statistical comparison of mean values in the RBE was performed using unpaired two-tailed *t*-test analysis. Error bars included in graphical figures represent SD.

All experiments were performed in accordance with institutional ethical guidelines. This work does not contain any studies with human or animal subjects. All cell lines were commercially available and experiments were performed according to institutional and national guidelines. An ethical approval was not needed for this type of study.

## Results

### Photon vs Carbon Ion Irradiation

**Figure 1:**
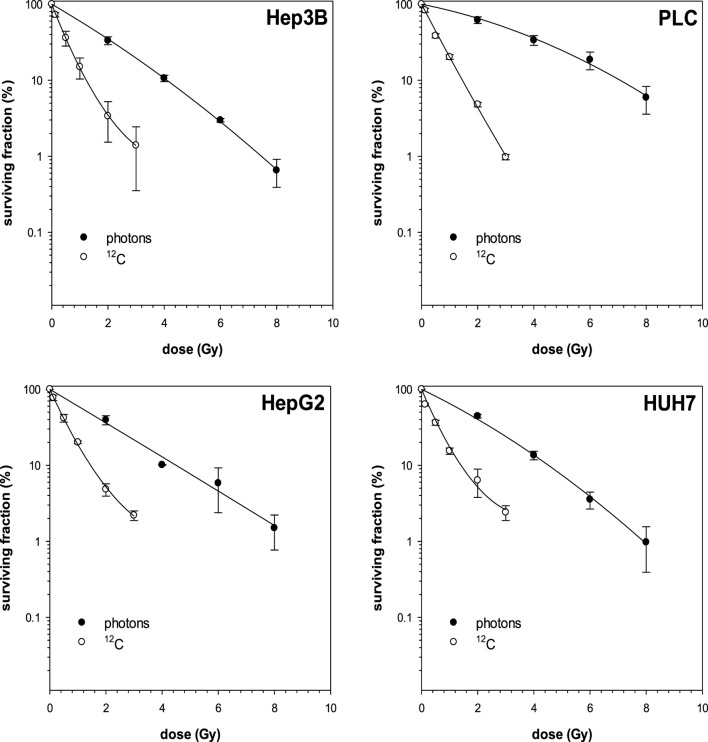
**Fitted survival curves of Hep3B and PLC cells after photon- and carbon ion-irradiation**. Abbreviations: ^12^C, carbon ion irradiation. Error bars represent SD.

First, photon and ^12^C irradiation was performed with all four HCC cell lines Hep3B, HepG2, PLC, and HUH7 to determine clonogenic survival. Clonogenic survival correlated negatively with increasing radiation doses for both RT modalities. The average numbers of PE for the cell lines in the control group were 8.1% for Hep3B, 6.3% for HepG2, 4.9% for PLC, and 13.6% for HUH7. The surviving fractions determined were used to perform linear-quadratic fits and calculate clonogenic survival curves for each cell line in order to compare the effectiveness of photon and ^12^C irradiation. All four cell lines showed a dose-dependent suppression of clonogenic survival as can be seen by typical shoulder shaped curves. The ^12^C dose-response curves showed a consistently steeper decrease of surviving cells with increasing doses compared to photons (*p* ≤ 0.01; Figure 1). All survival curves after X-ray irradiation follow the linear-quadratic model. However, some survival curves after ^12^C irradiation showed an upward tailing. The RBE values were calculated by at the 10%-survival level. The results are shown in Table 1. ^12^C irradiation showed an enhanced relative biological effectiveness toward clonogenic cell death induction than low-LET irradiation with photons. The comparison of RBE values at the 10%-survival level of photon and ^12^C irradiation ranged from 3.05 for HUH7 to 4.8 for PLC cells.

**Table 1:** **Calculated RBE values for 10% cell survival for all cell lines after photon- and carbon ion-irradiation**.

	Photons (Gy)	^12^C (Gy)	RBE at 10% SF
Hep G2	4.17161	1.34914	3.09
Hep 3B	4.1123	1.17415	3.50
HUH 7	4.56393	1.49346	3.05
PLC	7.05334	1.46768	4.80

*^12^C, carbon ion irradiation; RBE, relative biological effectiveness; SF, surviving fraction*.

### Treatment with either GEM or TEM

**Figure 2:**
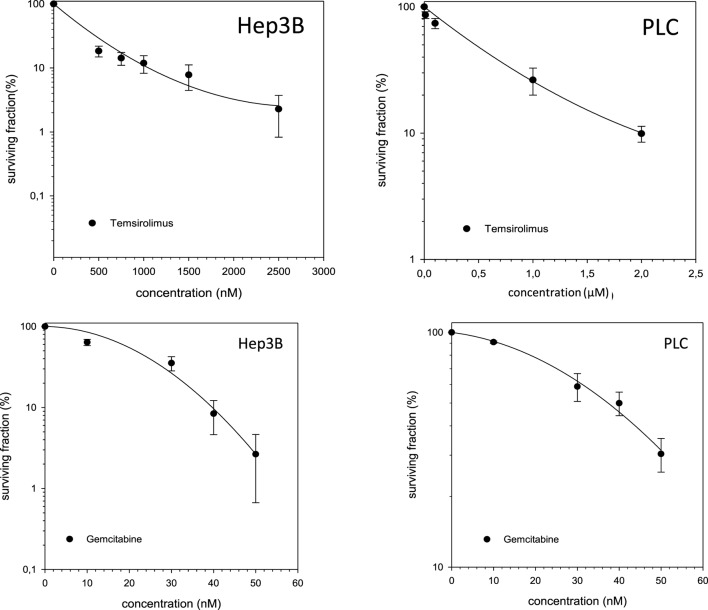
**Fitted survival curves of Hep3B and PLC cells after treatment with Gemcitabine (GEM) or Temsirolimus**. Abbreviations: ^12^C, carbon ion irradiation. Error bars represent SD.

Single-modality treatment of all cell lines with TEM or GEM led to a reduction of clonogenic survival in a dose-dependent manner. Table 2 summarizes calculated IC50 values for both substances in all four cell lines. IC_50_ concentrations for GEM and TEM were highest for HuH7 and Hep3B, respectively, and lowest for Hep3B and Huh7, respectively (Table 2). Figure 2 shows the survival curves of Hep3B and PLC cells after treatment with GEM or TEM. The surviving fractions determined were used to perform linear-quadratic fits and calculate clonogenic survival curves for each cell line in order to compare the cytotoxicity of GEM and TEM treatment.

**Table 2:** **IC_50_ values of GEM and Temsirolimus for all cell lines**.

	GEM (nM)	TEM (nM)
HepG2	20	190
Hep3B	19	210
HuH7	50	0.22
PLC	39	0.4

*GEM, Gemcitabine; TEM, Temsirolimus; IC_50_, half maximal inhibitory concentration*.

### Combined Treatment

**Figure 3:**
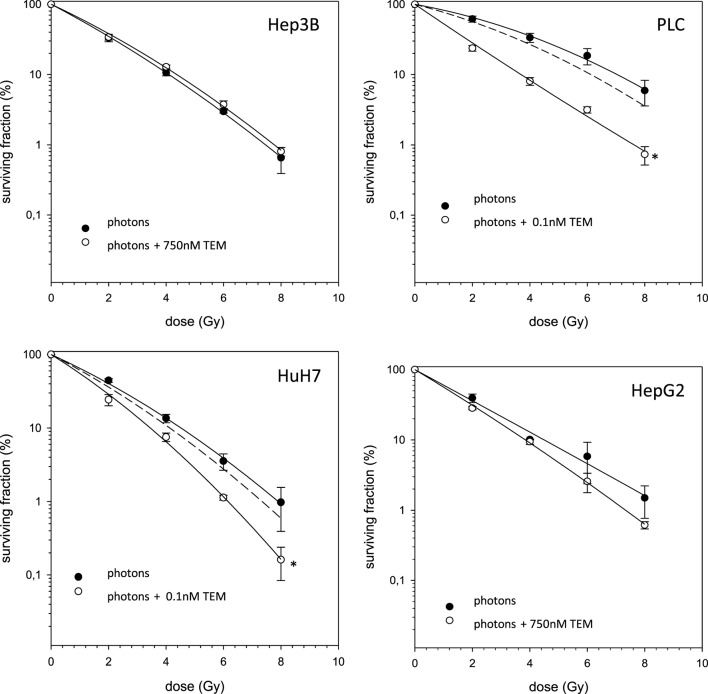
**Fitted survival curves of HepG2, Hep3B, HuH7, and PLC cells after combined modality treatment with photon irradiation and Temsirolimus**. Abbreviations: TEM, Temsirolimus; GEM, Gemcitabine. Error bars represent SD. The dashed line presents the predicted survival curve for TEM or GEM plus radiation, which was corrected for drug toxicity by normalizing the survival curve to the corresponding non-irradiated control group.

**Figure 4:**
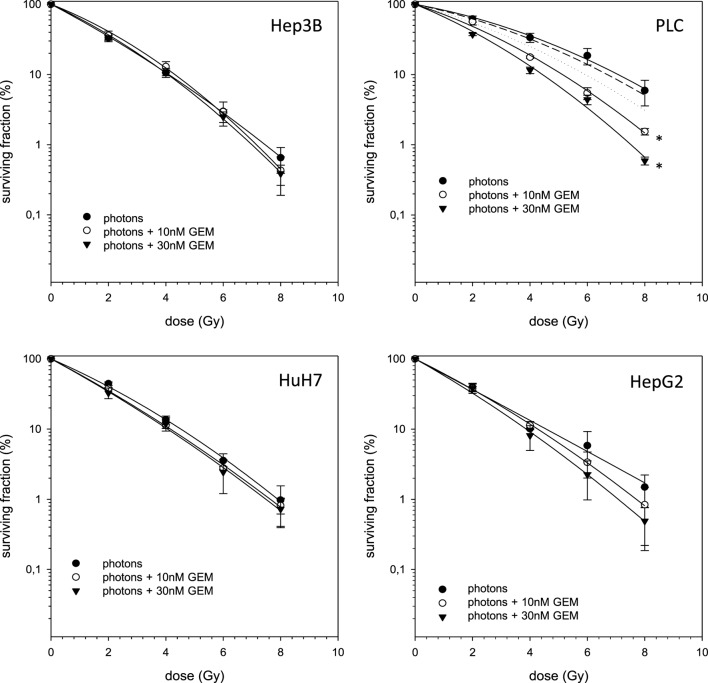
**Fitted survival curves of HepG2, Hep3B, HuH7, and PLC cells after combined modality treatment with photon irradiation and Gemcitabine**. Abbreviations: TEM, Temsirolimus; GEM, Gemcitabine. Error bars represent SD. Error bars represent SD. The dashed line presents the predicted survival curve for 10 nM GEM plus radiation, while the dotted line presents the predicted survival curve for 30 nM GEM plus radiation, which was corrected for drug toxicity by normalizing the survival curve to the corresponding non-irradiated control group.

In case of combination experiments with photon RT and TEM or GEM, PE values and averaged values were normalized to a drug control. Our experiments showed supra-additive cytotoxic effects for PLC cells (*p* ≤ 0.01) after irradiation and treatment with TEM or GEM (Figures 3 and 4). Supra-additive means that the combined effect is caused by lower doses of the two agents than is predicted. Furthermore, there were also supra-additive effects observed for HuH7 cells in combination of photon RT and TEM (*p* ≤ 0.01). All other combinations of drugs and photon irradiation showed independent toxicity in Hep3B, HepG2, and HUH7 cell lines.

**Figure 5:**
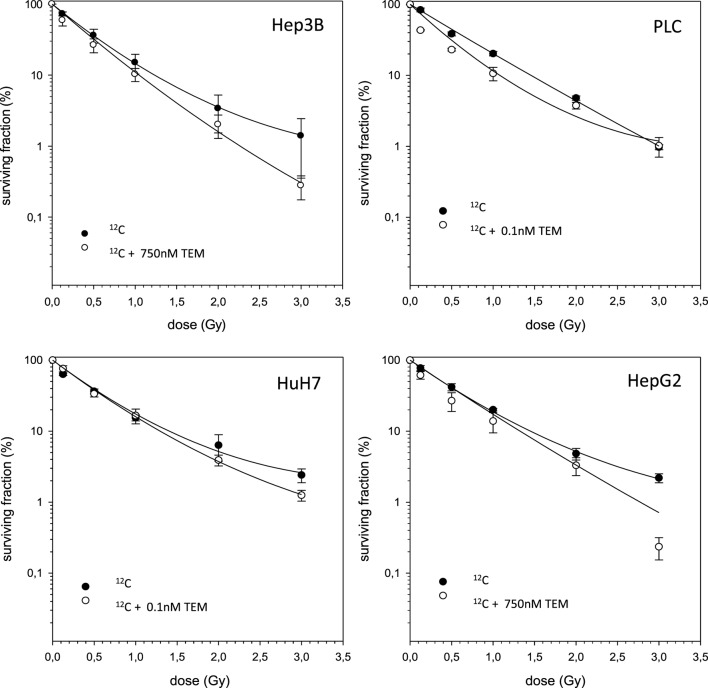
**Fitted survival curves of HepG2, Hep3B, HuH7, and PLC cells after combined modality treatment with ^12^C irradiation and Temsirolimus**. Abbreviations: TEM, Temsirolimus; GEM, Gemcitabine. Error bars represent SD.

**Figure 6:**
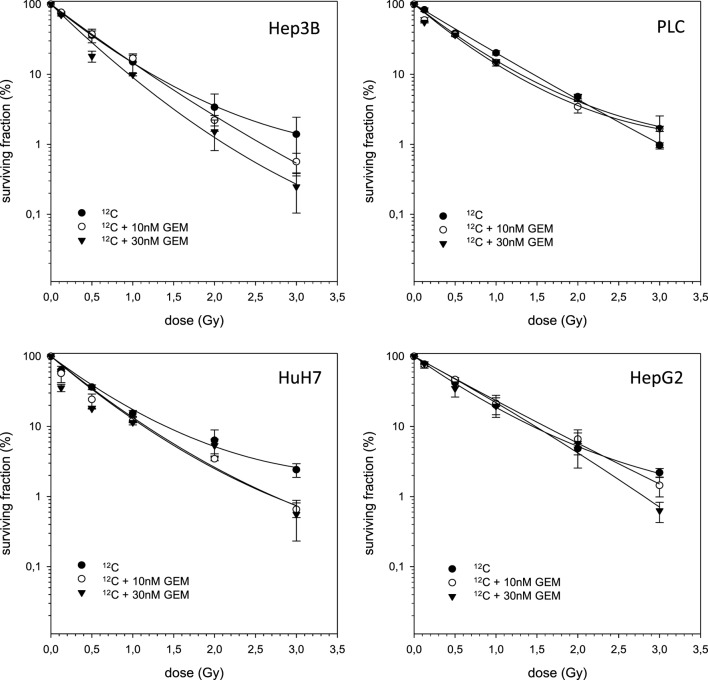
**Fitted survival curves of HepG2, Hep3B, HuH7, and PLC cells after combined modality treatment with ^12^C irradiation and Gemcitabine**. Abbreviations: TEM, Temsirolimus; GEM, Gemcitabine. Error bars represent SD.

The combined experiments of ^12^C irradiation together with TEM and GEM revealed independent toxicities and no additive or even supra-additive effects (Figures 5 and 6).

## Discussion

The results for the combination of ^12^C and both tested systemic substances TEM and GEM showed independent toxicities in all cell lines, whereas the combination of photon beams showed supra-additivity for the PLC cell line as defined using the isobologram method of Steel and Peckham (13). The same effect was seen for HuH7 cells treated with photon beams and TEM. In all other cell lines and combinations, only independent toxicities were observed. Some of the survival curves after ^12^C irradiation showed an upward tailing, which cannot be explained by the linear-quadratic mode. However, it is known that experimental data from high-LET radiation do not always fit to the linear-quadratic model (15). Using our special experimental setup, the results could not be merely explained as unattached mitotic cells not reached by high LET particles. It is known that high LET radiations increase also the complexity of lesions due to the formation of multiply damaged sites. However, the linear-quadratic model did not relate to this important aspect (15).

Until now, RT as a single modality has not played an important role for the treatment of HCC, since the liver tolerance to RT is poor (16). Many technical improvements in conformal RT, such as IMRT and PBT, have led to better dose distributions and warrant better sparing of the surrounding tissue (17). Many studies on SBRT and PBT in HCC patients demonstrated encouraging results (5, 17). With the implementation of PBT, ^12^C beams give hope of improved tumor responses to irradiation. Carbon ion beams have additional biological advantages compared to protons or X-rays, decreased oxygen enhancement ratio, reduced cell cycle-dependency and the potential of metastases suppression (18, 19). Because of its physical and biological advantages, ^12^C RT is a promising modality in the treatment of patients with HCC. This was confirmed by *in vitro* experiments of our group with rectal, pancreatic, and lung cancer cells showing that carbon beam ion beams exert a high RBE (20–23). Experimental data on the efficacy of heavy ions including ^12^C and ^16^O in HCC cell lines are encouraging. Recently, an enhanced RBE for ^12^C and ^16^O in these cell lines was shown by our group (24).

Several studies of ^12^C RT in HCC patients have been launched. Recently, preliminary results of a phase-I clinical trial evaluating ^12^C RT in HCC patients were reported (25, 26). Patients were irradiated with 4 × 10 Gy (RBE) without experiencing severe adverse effects during follow-up. No local relapse was observed during the follow-up period. Komatsu et al. performed a further study on 343 patients with 386 tumors of which 101 patients with 106 tumors received carbon ion therapy (17). Median follow-up was 31.0 months and the 5 years local control rates for patients receiving ^12^C treatment was 93%. Finally, available clinical studies on ^12^C RT in HCC patients demonstrate an overall safe and efficient therapy and may be an alternative to standard treatments a selected patient group.

Since treatment of locally advanced or metastatic HCC is still challenging and unsatisfactory, several systemic drugs have been tested for patients presenting with late stage disease. Among these there is GEM having shown broad activity against a variety of solid tumors, especially in hepatobiliary malignancies (11, 12, 27). GEM is a cytidine analog, which is phosphorylated to the active nucleotides gemcitabine diphosphate (dFdCDP) and triphosphate (dFdCTP). The anti-proliferative activity is mediated by various mechanisms. Preclinical studies demonstrated strong activity of GEM against HepG2 cells with an inhibition constant of about 3.98nM (28). This is consistent with the results of this work where an IC_50_ value is achieved at a concentration of around 20nM in HepG2 cells after an incubation period of 4 h. Therefore, it has to be taken into account that the incubation time of the experiments of Graziadei et al. was clearly longer (96 h) than ours. The IC_50_ values of our experiments using Hep3B, PLC, and HuH7 ranged from 19 to 50nM. Unfortunately, following phase II clinical trials with single agent GEM showed only marginal activity in HCC with response rates between 0 and 20% (29–31). Combination of photons and GEM revealed independent toxicities for all cell lines except for PLC cells, for which supra-additive effects were demonstrated.

Another developing strategy of treatment of advanced HCC is the therapy with molecular targeting drugs interfering with distinct pathways of carcinogenesis. TEM is one of them targeting mammalian target of rapamycin (mTOR) that is a part of the PI3K/Akt/mTOR-pathway, whose aberrant activation has been shown to be an important mechanism in the malignant transformation in HCC (9). This pathway is activated by binding of growth factors (i.e., EGFR, VEGFR, PDGFR, and IGFR) to the membrane receptor thus leading to PI3K-activation, which in turn downstream activates effectors such as Akt and mTOR. It could be shown that approximately 50% of HCC demonstrate aberrant mTOR activation (32). Inhibition of mTOR blocks key signal transduction pathways regulated by p70s6 and the eukaryotic initiation factor 4E-binding protein (4E-BP1). This does not only result in cell cycle arrest at G1 but also in reduced proliferation, impaired angiogenesis, and improved survival (32, 33). Moreover, TEM interacts with proteins like BAD, Bcl2, and p53 regulating apoptosis resulting in a shift of balance toward apoptosis. The four analyzed HCC cell lines showed different responses after treatment with TEM. HUH7 and PLC seem to be much more sensitive than Hep3B and HepG2 (IC_50_ = 0.22nM for HUH7; IC_50_ = 0.4nM for PLC; IC_50_ = 190nM for HepG2; IC_50_ = 210nM for Hep3B). Our findings are consistent with experiments by Zhou et al. in principle (34). They performed *in vitro* experiments with HUH7, Hep3B, PLC, and HepG2 and demonstrated IC_50_ values of 1.27μM for HUH7, 8.77μM for HepG2, 11.21μM for PLC, and 52.95μM for Hep3b after an incubation time of 24 h showing HUH7 to be the most sensitive cell line. Recently, two phase-I and -II studies on the efficiency of mToR-directed targeted substances Everolimus and Sirolimus showed only modest activity in advanced HCC (35, 36).

Since combination of TEM and RT in HCC has not been investigated yet, we performed *in vitro* studies on TEM with either photon or ^12^C RT. The combination of both showed supra-additive effects on HuH7 and PLC cells, whereas a small response was seen in Hep3B. HepG2 cells tendentially exhibit supra-additive effects on cell survival but due to overlapping SDs no clear point can be made. Therefore, we classified the observed effects as independent toxicity. Comparing this to monotherapy with TEM similar tendencies can be seen with PLC and HUH7 being the most sensitive cells, while Hep3B cells showed minimal effects. The results mentioned above lead to the conclusion that TEM radio-sensitizes HuH7 and PLC cells, whereas this effect was not observed for HepG2 and Hep3B cells.

Regarding the combination of ^12^C beams either with TEM or GEM, all cell lines exhibit independent toxicities. It can be assumed that combining ^12^C with systemic substances only has independent effects because heavy ions cause direct damage because of their high-LET character resulting in clustered DSBs. These DSBs are the most lethal molecular injuries that can be done to DNA, and therefore, addition of systemic drugs should not lead to radiosensitizing effects (20). Nonetheless, further investigations are warranted in order to determine whether addition of systemic therapy allows a reduction of radiation doses in combination therapy. This could possibly lead to better responses and tolerances in patients with HCC.

## Author Contributions

SD conducted most of the experiments, performed all cell survival calculations, and drafted a first manuscript version. CF helped to perform some of the experiments and helped to compute and analyze the experimental data. SB organized and helped to conduct the ion beam experiments. K-JW and TS helped to compute and analyze the experimental data. K-JW, SR, DB, and DH supervised the irradiation experiments. SR, SD, SB, K-JW, TS, JD, and SC helped to interpret the data and contributed to their analysis. DH conceived the study design, helped to analyze and interpret the data, and wrote the manuscript. TH, JD, and SC contributed with regard to scientific context and financial or technical support. SC conceived of the study and helped to finalize the manuscript. All authors helped with the interpretation of the data, revised the manuscript critically, and approved the final manuscript.

## Conflict of Interest Statement

The authors declare that the research was conducted in the absence of any commercial or financial relationships that could be construed as a potential conflict of interest.
